# The evolution of cervical spine trauma classification: a paradigm shift from morphological description to clinical decision-making

**DOI:** 10.3389/fneur.2025.1728720

**Published:** 2025-12-10

**Authors:** Xihao Huang, Yihong Zhang, Haowei Xiao, Jinlong Chen, Yu Jiang

**Affiliations:** Department of Orthopaedics, Peking University Third Hospital, Haidian, Beijing, China

**Keywords:** cervical spine trauma, spinal cord injury, classification system, spinal stability, Subaxial Injury Classification (SLIC), AO spine classification, paradigm shift

## Abstract

**Objective:**

This review systematically traces the evolution of subaxial cervical spine classification, highlighting the paradigm shift from morphological description to decision-oriented functional assessment and exploring future technological directions.

**Methods:**

A comprehensive narrative literature review was conducted, analyzing key classification systems, their underlying principles, and the technological advancements shaping the field.

**Results:**

Early mechanistic classifications were limited by poor interobserver reliability. The Subaxial Injury Classification (SLIC) system was a pivotal advance, integrating morphology, disco-ligamentous complex (DLC) integrity, and neurological status into a treatment-guiding score. However, its inconsistent reliability, particularly in DLC assessment, limited its adoption. The subsequent AO spine classification resolved these issues by introducing a more rigorous, hierarchical framework that achieved excellent, validated interobserver reliability. Crucially, the AO spine system also provides significant prognostic value by correlating morphological subtypes with long-term neurological recovery.

**Conclusion:**

The classification of cervical trauma has transitioned from a descriptive to an applied science. Future developments promise to resolve remaining challenges: artificial intelligence (AI) offers a definitive solution to interobserver variability, advanced imaging like diffusion tensor imaging (DTI) will refine prognostication, and brain-computer interfaces (BCI) provide new hope for functional reconstruction in severe injuries, heralding an era of precision medicine.

## 1 Introduction

Cervical spine trauma is a significant public health challenge in China, imposing a heavy burden on society due to its high rates of disability and mortality. The majority of those injured are middle-aged men between the ages of 40 and 69, with a male-to-female ratio of 4.43:1. The highest-risk occupational groups include farmers, workers, and drivers (1). In recent years, there has been a notable structural change in the causes of cervical spine injuries. Data indicates that from 2012 to 2019, the proportion of injuries resulting from falls increased significantly, rising from 47.1 to 64.9% (2). This shift has led falls to surpass traffic accidents as the primary cause of injury (which decreased from 45.3 to 22.6%). This transition is closely linked to an aging population, as the average age of patients who suffer fall-related injuries is noticeably higher than that of those involved in traffic accidents.

The clinical significance of cervical spine trauma is manifested in both the complexity of its management and the severity of its prognosis. Injuries involving the upper cervical spine (C1–C2) are often life-threatening, carrying a particularly high mortality risk. Conversely, trauma to the lower cervical spine commonly results in devastating neurological sequelae, including quadriplegia. The clinical course is frequently burdened by serious complications—especially respiratory infections, pressure injuries, and deep vein thrombosis—that are major determinants of mortality. In recognition of the distinct anatomical and biomechanical properties of the upper cervical spine, its classification systems are inherently independent and complex. This review, therefore, will primarily address the classification of the subaxial cervical segments.

A comprehensive understanding of the injury mechanism enables clinicians to better predict the potential and hidden risks of ligament and spinal cord injuries based on static imaging findings. An effective classification system translates this complex injury information into a standardized language. This not only facilitates academic communication among clinicians but, more importantly, directly informs treatment decisions. It helps assess the stability of the spine, determine the need for surgical intervention, and choose the appropriate surgical plan. These factors are crucial as they directly impact the recovery of neurological function and the long-term quality of life for patients. This article aims to uncover a fundamental cognitive shift by conducting an in-depth analysis of the biomechanical factors underlying cervical spine injuries. It will review the evolution of the cervical spine injury classification system and focus on contemporary classification standards. Additionally, the article will offer insights on optimizing the current system for practical use and explore potential future developments in classification systems, including the integration of artificial intelligence and dynamic evaluation, and look forward to the possible development direction of the future classification system, such as the integration of artificial intelligence, dynamic evaluation, advanced imaging technology, brain-computer interface (BCI) etc.

## 2 Biomechanics and mechanisms of cervical injury

The cervical spine is the most mobile, delicate, and complex part of the spine. Its biomechanics hinge on the balance between “mobility” and “stability.” This region must not only support the weight of the head, which represents approximately 8% of an adult’s body weight, but also allow for a wide range of flexible movements. These functions are vital for protecting the spinal cord and nerve roots contained within the cervical region. Therefore, understanding the biomechanics of the cervical spine, particularly its stability mechanisms, is crucial for the diagnosis and treatment of cervical injuries.

From an anatomical perspective, cervical stability is maintained collectively by bones, intervertebral discs, ligaments, and muscles. The lower cervical spine (C3–C7) bears compressive loads, with the facet joints limiting shear and rotational stresses. The intervertebral discs connect adjacent vertebral bodies, distribute stress, and allow for micro-motions between segments. Ligamentous structures act like cables that restrict excessive cervical motion. The anterior and posterior longitudinal ligaments limit hyperextension and hyperflexion, respectively, while the posterior ligamentous complex (PLC)—located posterior to the vertebral bodies and comprising the supraspinous ligament, interspinous ligament, and ligamentum flavum—forms a “posterior tension band” that resists flexion stress. The integrity of the PLC is essential for maintaining sagittal alignment of the cervical spine and preventing progressive deformity. According to Panjabi’s spinal stability theory (3), these passive structures collectively define the “neutral zone” of spinal motion. Injury to passive structures leads to an enlarged neutral zone, which serves as a sensitive indicator of instability. In contrast, muscles can actively reduce the neutral zone through contraction, effectively compensating for deficits in passive stability. This mechanism underscores the critical role of muscles in dynamic stabilization.

Scholars have proposed various theoretical models for this purpose, among which the “two-column theory” and the “three-column theory” have had the most significant impact. These theories are fundamental to understanding the critical issue of “stability” following spinal trauma. In 1970, Holdsworth (4) introduced the “two-column theory,” marking a revolutionary approach in evaluating spinal stability. Prior to this, descriptions of fractures primarily focused on their morphology. According to this theory, the spine can be functionally divided into two columns. The anterior column consists of the anterior longitudinal ligament, vertebral body, intervertebral disc, and posterior longitudinal ligament, which primarily bear compressive loads. In contrast, the posterior column includes all bony structures and ligaments at the back, which mainly resist tension. Holdsworth proposed that the stability of the spine relies entirely on the integrity of the posterior column, particularly the posterior longitudinal ligament (PLC). This perspective shifts the clinical focus from merely assessing fracture morphology to evaluating the integrity of this key stabilizing structure. This change directly influences treatment decisions and lays the groundwork for the development of subsequent theories.

To address the limitations of the two-column theory, Denis (5) proposed the three-column theory in 1983, based on an analysis of a large number of CT scans. He divided the spine into three columns: The anterior column, which includes the anterior longitudinal ligament, the anterior half of the vertebral body, and the intervertebral disc. The middle column, which consists of the posterior half of the vertebral body and intervertebral disc, as well as the posterior longitudinal ligament. The posterior column, which encompasses the pedicle and all posterior structures behind it. A key contribution of the three-column theory is the introduction of the “middle column” concept. This column acts as a mechanical hub connecting the anterior and posterior columns, making its integrity crucial for stability. This model significantly improves the accuracy of assessing instability in complex injuries, such as burst fractures, which are typical injuries involving the middle column. It provides a more precise and reliable basis for making surgical decisions.

However, the three-column theory has inherent limitations (6). Firstly, it remains a mechanical model that primarily focuses on bone structure injuries. In an era with only X-ray and CT imaging, the assessment of the posterior ligament complex (PLC), which involves pure soft tissue injuries, still relies on indirect signs, leading to assessments that are not always intuitive or accurate. Secondly, the theory has limited capability in evaluating unstable damage resulting from complex mechanical mechanisms such as rotation and shear. Finally, similar to the two-column theory, it does not address neurological status.

## 3 Main injury mechanics modes and typical injury conditions

### 3.1 Compressive injuries

**Figure 1:**
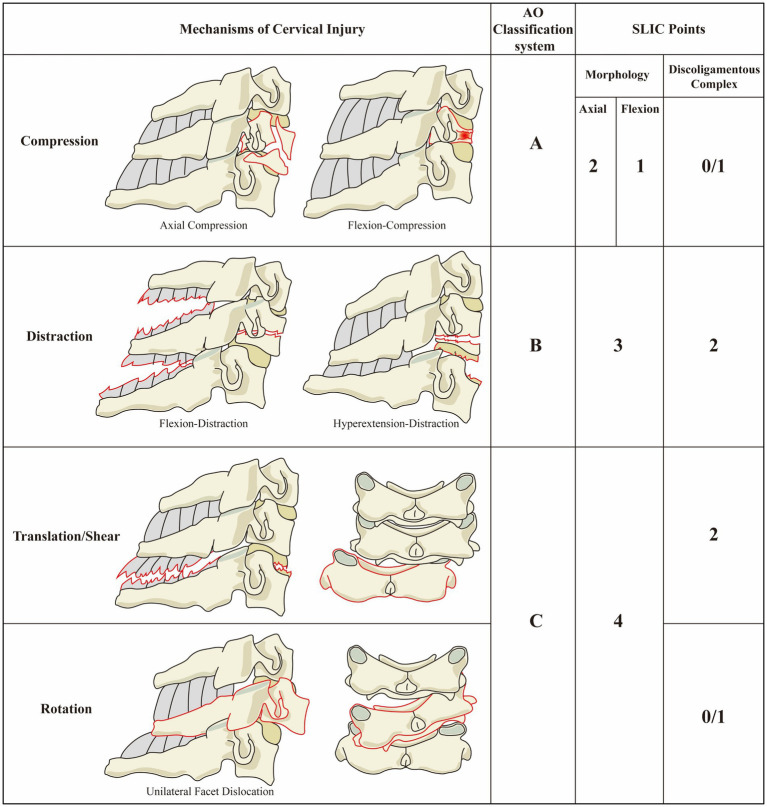
Three main injury mechanics modes (14, 19), adapted from Vaccaro et al. (18).

Violence to the cervical spine can be categorized into two main types based on the direction of the force: (1) Axial compression: This occurs when an external force is applied vertically downward along the long axis of the cervical spine while in a neutral position. Common causes include falls from a height or axial injuries, such as those sustained during diving incidents. In this case, the vertical pressure ensures that stress is evenly distributed across the vertebral body. However, if the force exceeds the structural limits of the bones, it can lead to burst fractures, where the vertebral body breaks apart. The fracture fragments often displace backward, invading the spinal canal, which can result in severe spinal cord injury. (2) Flexion compression: This type of injury occurs when there is a combination of axial compression and forward flexion forces. The stress concentrates at the front of the vertebral body, causing the anterior column to compress and collapse. Meanwhile, the middle column remains relatively intact or only slightly compressed, and the ligaments of the posterior column are either intact or only slightly strained. This usually results in wedge compression fractures, which are characterized by a wedge-shaped deformation of the vertebral body, featuring a lower anterior region and a higher posterior region (Figure 1).

### 3.2 Distraction injuries

Distraction injuries occur when two adjacent vertebral bodies are pulled apart along their long axis, leading to the tearing and separation of ligaments or bony structures. In this type of injury, the front column is compressed, while the middle and rear columns (particularly the posterior ligament complex, or PLC) are subjected to enormous tension, causing them to “tear apart.” This results in a highly unstable injury because the PLC, functioning as the “posterior tension band,” becomes ineffective. Consequently, this can lead to bilateral dislocation of facet joints or horizontal fractures that penetrate the vertebral body, such as a Chance fracture (Figure 1).

In cases of hyperextension traction, as opposed to flexion traction, there is extreme retroversion of the neck. This causes compression of the posterior column structures, such as spinous processes and laminae, which then act as a rotating fulcrum. At the same time, the anterior structures (including the anterior longitudinal ligament and the front part of the intervertebral disc) are subjected to strong tension and can tear. This may result in a tear of the intervertebral disc or an avulsion fracture at the anterior and inferior edge of the vertebral body. In elderly patients with spinal stenosis, this mechanism can lead to severe central spinal cord syndrome (7) due to compression of the ligamentum flavum, even if no fractures are present (Figure 1).

### 3.3 Translation/shear and rotation injuries

Displacement/shear force acts directly in the horizontal direction of the spine, parallel to the vertebral endplate (8). It causes the upper vertebral body to slide horizontally relative to the lower vertebral body. This type of injury mechanism is high-energy force and typically indicates severe damage to the three-column structure of the spine, leading to catastrophic spinal instability and potential spinal cord injury. When an external force includes a torsional component, it can result in rotational damage. The rotational force has a unique destructive effect on the facet joints and their surrounding joint capsules that normally restrict rotation. When this rotational force is combined with a flexion force (known as flexion-rotation), it often leads to unilateral facet dislocation. This occurs when one facet “jumps over” and becomes “stuck” in front of the adjacent facet, resulting in an interlocking situation (6) (Figure 1).

## 4 Evolution of subaxial cervical spine classification systems

### 4.1 Early exploration of the classification system

**Figure 2:**
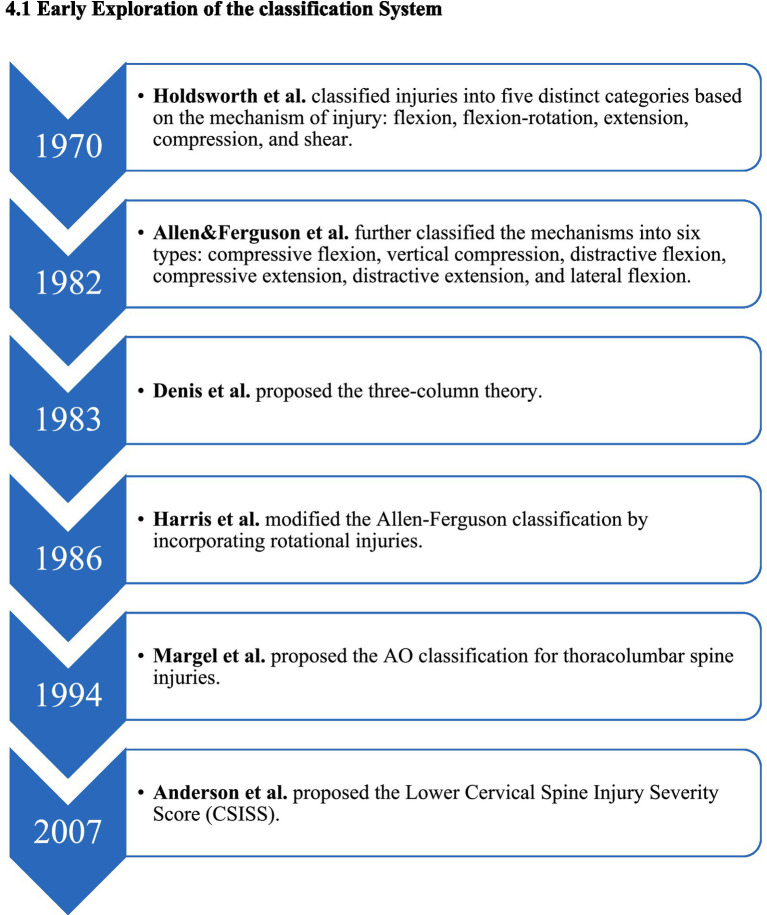
Evolutionary timeline of the early subaxial cervical spine classification system.

The classification system for lower cervical spine injuries has evolved from basic morphological descriptions to more comprehensive evaluations of injury mechanisms, spinal stability, nerve function, and clinical treatment guidance. This evolution reflects advancements in imaging technology, a deeper understanding of biomechanics, and the rise of evidence-based clinical medicine (Figure 2).

In 1970, based on clinical experience and X-ray findings, Holdsworth (4) functionally divided spinal injuries into anterior and posterior columns. This marked a revolutionary shift in understanding, emphasizing that the integrity of the posterior column is crucial for maintaining spinal stability. He classified damage into five mechanisms: buckling, buckling/rotation, extension, compression, and shear. Although this classification is not exhaustive, it laid the theoretical groundwork for evaluating “stability,” and its influence persists today.

In 1983, Denis et al. (5) built upon Holdsworth’s work by analyzing numerous CT scans and proposing the influential three-column theory, primarily for the thoracolumbar spine. He introduced the concept of the “middle column” and stated that “two or more column injuries indicate instability.” While not specifically designed for the cervical spine, this theory has strong explanatory power and practicality, significantly impacting the assessment of stability in spinal trauma.

In 1982, Allen et al. (14) classified cervical spine injuries into six main types based on the position of the cervical spine and direction of the applied force: flexion-compression, vertical compression, flexion-stretch, extension-compression, extension-stretch, and lateral flexion. Each type details varying stages of subaxial cervical spine injury, indicating that injuries at higher levels result in greater anatomical instability.

In 1986, Harris et al. (19) enhanced the Allen and Ferguson classification by incorporating rotation injuries to more accurately explain and categorize injuries caused by complex forces. In 1994, Margel et al. (9) introduced the thoracolumbar AO classification, which is primarily based on pathomorphology and systematically increases in severity. This classification divides spinal fractures into three categories: A (compression), B (distraction), and C (rotation/displacement), each with further subtypes. While successful in classifying thoracolumbar fractures, its application to lower cervical spine injuries is limited due to its complexity, consisting of over 30 subtypes, and its poor adaptability to the unique anatomical features and injury mechanisms of the lower cervical spine.

In 2007, Anderson et al. (10) proposed the Lower Cervical Spine Injury Severity Score (CSISS) to quantify injury severity. This system divides the cervical spine into four columns—anterior, posterior, left, and right—scoring each separately and aggregating the results. A total score exceeding 7 indicates a recommendation for surgery. The CSISS has the advantage of evaluating ligament injuries and stability quantitatively; however, the scoring can vary between doctors, and it does not account for neurological status, limiting its widespread adoption (13).

In summary, earlier theories have provided a fundamental understanding of bone structure damage viewed as a “pillar.” With advancements in MRI and other imaging technologies, we can now visualize damage to the posterior ligament complex (PLC) as a “cable.” Moreover, surgical treatment approaches and evidence-based medicine have shown that PLC injuries significantly affect prognosis. Consequently, PLC has evolved from being a neglected component in past theories to a focal point in modern spinal injury assessments, aligning with bone structure evaluations. More refined classification systems, such as SLIC and the AO spine scoring system, now incorporate PLC as an independent and high-weight variable, allowing for more accurate clinical treatment guidance.

### 4.2 Subaxial cervical spine injury classification system (SLIC)

The system proposed by Vaccaro et al. (12) marked a significant advancement in the assessment of cervical spine injuries. It was the first to incorporate both the intervertebral disc-ligament complex and the patient’s neurological status into a scoring system. The major innovation of this system is the move away from complex morphological descriptions to an integrated scoring approach that focuses on three core elements influencing prognosis and treatment, establishing a direct correlation with clinical decision-making: morphology (categorized based on severity into four types: compression, burst, stretch, and rotation/displacement), disco-ligamentous complex (DLC) [the integrity of soft tissues, including the posterior ligament complex (PLC)], and neurological status (the impact of the injury on the patient’s nervous system) (Table 1).

**Table 1:** SLIC scale, adapted from Vaccaro et al. (12).

Category	Item/criteria		Points
Morphology	No abnormality		0
	Compression		1
		Burst	+1 = 2
	Distraction (e.g., facet perch, hyperextension)		3
	Rotation/translation (e.g., facet dislocation, unstable teardrop or advanced staged flexion compression injury)		4
Disco-ligamentous complex (DLC)	Intact		0
	Indeterminate (e.g., isolated interspinous widening, MRI change only)		1
	Disrupted (e.g., widening of disc space, facet perch or dislocation)		2
Neurological status	Intact		0
	Root injury		1
	Complete cord injury^a^		2
	Incomplete cord injury^a^		3
	Continuous cord compression in setting of neuro deficit (neuro modifier)		+1

a Higher score reflects greater potential for neurological recovery, thus higher urgency for intervention, lower score reflects limited potential for recovery.

The SLIC (Subaxial Injury Classification) assigns scores as follows: a total score of 1–3 points suggests conservative treatment, a score of 5 or more points indicates the need for surgical intervention, and a score of 4 points represents a critical state. The primary advantages of SLIC are its simplicity, high reliability, and reproducibility. Importantly, it directly links classification to treatment plans for the first time, providing clear and quantitative guidance for clinical decisions (15). Consequently, it has gained widespread adoption as the leading classification system for lower cervical spine injuries globally (Table 1).

The score for incomplete injuries (3 points) is higher than that for complete injuries (2 points). This is because patients with incomplete injuries have greater recovery potential and typically require more urgent surgical intervention to preserve neurological function (Table 1).

Since the introduction of SLIC, researchers have proposed several new typing systems. Notably, Patel et al. (15), Tsou et al. (16), and Shousha et al. (17) each developed their own schemes. However, these subsequent typing systems have not been widely adopted in clinical practice, primarily due to their low reliability or their failure to demonstrate advantages over SLIC.

### 4.3 AO classification system

In 2016, the American Spine Injury Classification (ASIC) system introduced the lower cervical spine injury classification system based on the SLIC scoring system (18). This system aims to provide a more comprehensive, concise, and reliable classification standard to enhance clinical communication and support scientific research. It is built on the successful experiences of the AO classification in interpreting thoracolumbar trauma, focusing on breaking down complex injury patterns into a thorough evaluation of several key dimensions. The framework of the ASIC system consists of four injury types: Type A (compression injuries) involves compression injuries where the posterior tension band remains intact, primarily manifesting as vertebral compression fractures. Type B (tension band injuries) describes tension band damage, which occurs due to fracture of the posterior or anterior tension band structure (ligament or bone) caused by tension forces, without any displacement of the vertebral body. Type C (translational injuries) is the most severe type, defined by significant displacement or dislocation of the vertebral body in any direction, indicating a complete loss of stability in the spinal structure. Type F (facet injuries) specifically addresses small joint injuries, ranging from F1 (small joint fracture without displacement) to F4 (articular process dislocation or interlocking), accurately depicting various degrees of injury to this critical stable structure (Table 2).

**Table 2:** AO spine subaxial cervical injury classification system, adapted from Vaccaro et al. (18).

Injury type	Injury mechanism	Subtype	Specific characteristics
Type A	Compression injuries (vertebral body compression with intact tension band)	A0^a^	Minor, non-structural injuries (e.g., isolated spinous process, transverse process, or lamina fracture); also includes central cord syndrome without radiographic evidence of fracture
		A1	Wedge-compression fracture involving a single endplate, without involvement of the posterior wall of the vertebral body
		A2	Split or pincer fracture involving both endplates, without involvement of the posterior wall of the vertebral body
		A3^b^	Incomplete burst fracture: involvement of a single endplate and the posterior wall of the vertebral body
		A4^b^	Complete burst fracture: involvement of both endplates and the posterior wall; OR a sagittal split fracture involving the posterior wall
Type B^c^	Tension band injuries (failure of anterior or posterior tension band with maintained spinal alignment)	B1	Bony posterior tension band failure (e.g., chance-type fracture through bone)
		B2	Complete posterior capsuloligamentous or osseo-ligamentous disruption
		B3	Anterior tension band failure (hyperextension injury) traversing the disc or vertebral body, with an intact posterior hinge preventing gross displacement
Type C	Translation/Displacement injuries (displacement/translation of one vertebral body relative to another in any direction)	C	Any injury with clear translation or dislocation in any plane (anterior, posterior, lateral, or vertical distraction). This type can be superimposed on any Type A or B injury pattern
Type F	Facet injuries (injuries specifically involving the facet joint complex)	F1	Non-displaced facet fracture (superior or inferior articular process); fragment <1 cm and <40% of the lateral mass
		F2	Potentially unstable facet fracture; fragment >1 cm and >40% of the lateral mass, or displaced
		F3	Floating lateral mass: concurrent fractures of the ipsilateral pedicle and lamina, disconnecting the lateral mass
		F4^d^	Pathological subluxation or dislocation: includes “perched” facets (tip-to-tip alignment) or complete facet dislocation (“jumped” and locked)

a A0 is also used for patients with central cord syndrome but no fracture on CT, highlighting the importance of the neurological (N) score in these cases.

b Burst fracture. Involves the posterior vertebral wall, implying potential for canal compromise and neurological injury.

c Tension band injury (distraction). Assumed to be stable against translation, but grossly unstable in flexion or extension. If any translation is present, it is upgraded to a Type C.

d Grossly unstable. Signifies complete disruption of the disco-ligamentous complex at the facet joint.

To ensure a comprehensive description of each injury, the AO spine system also incorporates two important supplementary dimensions: neurological status and modifying factors. This approach creates a complete system composed of “main classification + supplementary classification.” Neurological status ranges from N0 (intact neurological function) to N4 (complete spinal cord injury), and includes NX (unable to assess), N + (persistent spinal cord compression), along with other special conditions. In addition, patient-specific modifying factors (M, modifiers) are factors that significantly influence clinical decision-making and enrich the clinical information conveyed by the classification. They also alert clinicians to potential risks that could alter treatment strategies. The modifying factors include: suspicion of injury to the posterior ligament complex (M1), dangerous disc herniation (M2), presence of underlying conditions such as metabolic bone disease or ankylosing spondylitis (M3) and evidence of vertebral artery injury (M4) (Table 3).

**Table 3:** Neurological status and clinical specific factors, adapted from Vaccaro et al. (18).

Category	Modifier	Specific characteristics
Neurological status (N)	NX	Neurology undetermined (used to designate patients who cannot be examined due to head injury or another condition which limits their ability to complete a neurological examination such as intoxication, multiple trauma, or intubation/sedation)
	N0	Neurologically intact
	N1	Transient neurological deficit
	N2	Radiculopathy
	N3^a^	Incomplete spinal cord injury
	N4^a^	Complete spinal cord injury
	+	Indicates ongoing cord compression in a patient with neurological deficits, signifying an urgent need for surgical decompression
Clinical specific factors (M)	M1	posterior capsuloligamentous complex injury without complete disruption
	M2	Critical disc herniation
	M3	Metabolic bone disease affecting stability [e.g., diffuse idiopathic skeletal hyperostosis (DISH), ankylosing spondylitis (AS)]
	M4	Signs of vertebral artery injury

a N3 (Incomplete SCI): Higher potential for neurological recovery. N4 (Complete SCI): Limited potential for recovery.

In conclusion, the AO spine lower cervical spine injury classification system offers a clear, structured, and logical framework for categorizing injuries. It utilizes a four-dimensional evaluation model that includes “morphology main classification, facet joint classification, neurological function classification, and correction factors.” By building upon the integrated concept of the Subaxial Cervical Spine Injury Classification (SLIC), this system provides a more systematic and detailed division of injury types. Its goal is to enhance the consistency and clinical applicability of the classification.

### 4.4 Comparison and integrated application of various systems

The evolution of cervical spine trauma classification systems reflects a fundamental shift from subjective inferences of injury mechanisms toward objective, reliable, and clinically practical morphological classifications. A comparative analysis of the traditional Allen–Ferguson system, the Subaxial Injury Classification (SLIC) system, and the AO spine classification reveals a clear developmental trajectory. Table 4 presents a comprehensive literature review exploring the comparison of classification systems.

**Table 4:** Comprehensive literature review of subaxial cervical spine trauma classification systems.

No.	Author	Title	Journal	Year	Main content
(13)	Whang et al.	The development and evaluation of the Subaxial Injury Classification scoring system for cervical spine trauma	Clinical Orthopaedics and Related Research	2011	An early evaluation by the SLIC developers. It reported that the overall reliability of SLIC (ICC ranged from 0.49 to 0.90) appeared at least as good as conventional schemes (ICC 0.41 to 0.53)
(15)	Patel et al.	Classification and surgical decision making in acute subaxial cervical spine trauma	Spine	2010	A systematic review that identified the SLIC system as the optimal classification scheme at the time, highlighting its integration of morphology, DLC, and neurology to guide treatment
(23)	Stone et al.	Reliability of classification systems for subaxial cervical injuries	Evidence-Based Spine-Care Journal	2010	Simultaneously evaluated CSISS, SLIC, and Allen–Ferguson (A&F) systems. Found excellent interobserver reliability for the total SLIC score (ICC = 0.79), while A&F showed moderate (kappa = 0.50) to poor (kappa = 0.34) reliability
(25)	Lee et al.	Interobserver and intraobserver reliability of sub-axial injury classification and severity scale	Journal of Korean Neurosurgical Society	2012	Assessed SLIC reliability among different specialists. Found substantial interobserver agreement for morphology (ICC = 0.603) and total score (ICC = 0.775), but only fair agreement for the disco-ligamentous complex (DLC) (ICC = 0.304)
(24)	van Middendorp et al.	The subaxial cervical spine injury classification system: an external agreement validation study	The Spine Journal	2013	A key external validation study that found poor interobserver agreement for the morphologic component of SLIC (kappa = 0.29) and moderate agreement for the DLC (kappa = 0.46), concluding that its reproducibility was limited due to ambiguity
(22)	Joaquim et al.	Evaluation of the Subaxial Injury Classification system	Journal of Craniovertebral Junction and Spine	2011	Focused on the clinical validity of the SLIC score in guiding treatment. Found a high concordance rate (over 91%) between the treatment predicted by the SLIC score and the actual treatment performed, suggesting it is a promising decision-making tool
(27)	Feuchtbaum et al.	Subaxial cervical spine trauma	Current Reviews in Musculoskeletal Medicine	2016	A review article that noted the poor interobserver reliability of SLIC’s morphologic classification and presented the newer AO spine system as a more reliable alternative, citing its robust interobserver (kappa = 0.64) and intraobserver (kappa = 0.75) agreement
(20)	Urrutia et al.	A comparative agreement evaluation of two subaxial cervical spine injury classification systems: the AO spine and the Allen and Ferguson schemes	European Spine Journal	2016	A direct comparative study demonstrating that the AO spine classification has significantly better interobserver agreement (kappa = 0.61) than the Allen–Ferguson classification (kappa = 0.46)
(29)	Urrutia et al.	An independent inter- and intraobserver agreement evaluation of the AO spine subaxial cervical spine injury classification system	Spine	2017	An independent validation of the AO spine system. It confirmed substantial interobserver agreement for the main fracture types (A, B, C, F) with a kappa value of 0.61, and moderate agreement for subtypes (kappa = 0.57)
(30)	Karamian et al.	An international validation of the AO spine Subaxial Injury Classification system	European Spine Journal	2023	A large-scale international validation (203 participants) establishing the AO spine system as highly reliable. It reported excellent interobserver reliability for fracture morphology (kappa = 0.87) and substantial reliability for fracture subtype (kappa = 0.80)
(28)	Schnake et al.	AO spine classification systems (subaxial, thoracolumbar)	Journal of Orthopaedic Trauma	2017	An overview of the then-new AO spine classification systems, outlining their design principles and reporting early, substantial reliability data for the subaxial system (kappa = 0.64)
(31)	Mushlin et al.	AO spine subaxial cervical spine injury classification system: the relationship between injury morphology, admission injury severity, and long-term neurologic outcome	World Neurosurgery	2019	This study validated the prognostic value of the AO spine classification. It demonstrated that morphological subtypes are significantly associated with admission injury severity and long-term neurologic outcomes, with A3/A4 types having the worst prognosis
(32)	Canseco et al.	The subaxial cervical AO spine injury score	Global Spine Journal	2022	Based on a global surgeon survey, this study developed the subaxial cervical AO spine injury score (AOSIS). It assigns specific point values to each AO spine classification variable to quantify injury severity, aiming to create a foundation for a surgical algorithm
(33)	Thumbadoo et al.	Dynamic radiographs in assessing stability of cervical spine fractures: a multicentre study	JAAOS Global Research & Reviews	2022	A critical study demonstrating the limitations of static, supine CT imaging. It showed that dynamic radiographs can reveal instability missed on initial scans, leading to a significant increase in the SLIC score (mean score changed from 0.73 to 6)
(26)	Shaharudin et al.	The initial assessment and management of cervical spine injuries: a comprehensive review	Cureus	2025	A comprehensive review covering the management of cervical spine injuries. It discusses both SLIC and AO spine classifications, noting SLIC’s reliance on MRI versus AO spine’s basis on CT as a key difference in clinical application

While the Allen-Ferguson system provided an intuitive, mechanism-based framework valuable for teaching and conceptualizing injuries, its reliance on subjective inferences has long been hampered by low interobserver reliability, rendering it an inadequate tool for standardized communication and modern clinical research (20, 21). The introduction of the Subaxial Injury Classification (SLIC) system marked a significant milestone. It was the first system to quantify three key variables—injury morphology, the integrity of the disco-ligamentous complex (DLC), and neurologic status—into a summated score linked to specific treatment thresholds, thereby shifting the paradigm from mere description to therapeutic guidance, with studies later showing its final score aligned well with actual treatment decisions (15, 16, 22).

However, the reliability of SLIC soon became a concern. Early evaluations noted considerable variability in its intraclass correlation coefficients (ICCs), which ranged from 0.49 to 0.90, with the lowest reliability observed in the assessment of the DLC (15). While some independent research reported excellent reliability for the total score (ICC = 0.79) (23), a pivotal external validation study confirmed significant weaknesses, finding poor interobserver agreement for morphology (kappa = 0.29) and only moderate agreement for the DLC (kappa = 0.46) (24). This identified weakness in DLC assessment was corroborated by another study, which found only fair agreement among different specialists (ICC = 0.304) (21, 25). Its reliance on MRI, which could delay decision-making, further limited its widespread adoption (26).

To address these reliability issues, the AO spine classification system was developed (27, 28). Building upon the core principles of SLIC, it introduced a more rigorous, hierarchical morphological classification that significantly improved reliability (28). Studies demonstrated that its interobserver agreement (kappa = 0.61) was significantly superior to that of the Allen–Ferguson system (kappa = 0.46) (20). Independent validation later confirmed its substantial reliability (29), and a large-scale international study ultimately established it as the gold standard, demonstrating excellent interobserver agreement (kappa = 0.87) (30). Crucially, the AO spine system also provides significant prognostic value. Its morphological subtypes correlate strongly with initial injury severity and long-term neurological recovery. For instance, A3/A4 (burst) fractures are associated with the poorest neurological outcomes, whereas C-type (translational) injuries show greater potential for recovery (31).

Furthermore, the AO spine classification directly addresses applicability in special cases via its specific modifiers (M-factors), which add crucial clinical context beyond osseous morphology. For instance, the M3 modifier redefines stability in patients with ankylosing spondylitis, while the M2 modifier prioritizes a critical disc herniation for surgical planning (19). The development of the AO spine injury score, by assigning significant quantitative weight to these modifiers, formally integrates the assessment of special populations into the classification to guide treatment (32).

In integrated clinical practice, the simplicity of the SLIC score remains valuable for rapid initial assessments. However, the superior reliability and proven prognostic utility of the AO spine system make it the preferred tool for complex case planning, academic communication, and clinical research. A critical limitation, however, applies to all systems based on static, supine CT imaging. A key study warned that the absence of gravitational loads in the supine position can mask dynamic instability, leading to underestimation of injury severity and misguided treatment decisions (33). Consequently, dynamic imaging is essential for patients with persistent clinical symptoms despite stable findings on CT (33).

In conclusion, clinical decision-making should not rely on a single classification system. While these systems are invaluable tools, they do not replace clinical judgment (26). An optimal management strategy requires the integration of radiographic classifications with dynamic functional assessments and the patient’s overall clinical presentation to formulate a safe and effective individualized treatment plan.

## 5 Special considerations and neurological assessment

The term “spinal cord injury without radiographic abnormality” (SCIWORA) was first conceptualized in the 1980s by Pang and Wilberger to describe pediatric patients with traumatic myelopathy despite normal X-rays showing no fracture or malalignment (34). While this phenomenon is predominantly seen in children and adolescents, the concept was later extended to adult patients (35). Its pathophysiological basis, especially in children, is attributed to the unique biomechanical properties of the pediatric spine, including the high elasticity of the intervertebral disc and ligamentous complex, the horizontal orientation of facet joints, and a relatively large head-to-trunk ratio. These factors allow for transient, severe spinal displacement during excessive flexion, extension, or distraction forces, which can cause significant internal spinal cord damage without resulting in permanent osseous injury (36–38).

The advent of magnetic resonance imaging (MRI) has profoundly evolved the understanding of SCIWORA. MRI allows clinicians to visualize soft tissue structures, revealing that many patients initially diagnosed with SCIWORA actually have definitive pathological changes such as spinal cord edema, contusion, hemorrhage, or ligamentous tears, a finding confirmed by systematic review (39–41). To better describe these findings, more precise terminology has been developed. For the rare cases where even high-resolution MRI shows no abnormality, the term “Real SCIWORA” has been proposed (42). In adults, where these injuries are often associated with underlying degenerative disease, terms like “spinal cord injury without traumatic imaging evidence” (SCIWITIE) are preferred to more accurately reflect the distinct pathophysiology (43).

In modern clinical practice, SCIWORA is a diagnosis of exclusion. Following practice guidelines, any child with post-traumatic neurological symptoms but normal X-ray/CT scans must undergo an MRI (44–46). This necessity presents a challenge to bony-injury-based classifications like the AO spine system. Because CT scans are normal, applying the standard morphological grading (A, B, C) is not applicable or can be misleading, as an “A0” classification would severely underestimate the injury’s true severity. Therefore, for a patient with SCIWORA, the clinical application of such classification systems must pivot away from the non-informative morphological grading. Instead, the focus shifts entirely to the neurological status (N-score) and the MRI findings, which are used to assess the integrity of the disco-ligamentous complex and spinal cord parenchyma. This comprehensive assessment, rather than the bony classification alone, dictates the true severity and guides management. MRI is not only the gold standard for diagnosis but also the most important tool for prognosis (34). For patients with SCIWORA but without structural instability confirmed by MRI, conservative treatment such as cervical collar fixation is the standard of care. Notably, even “Real SCIWORA” patients who achieve full neurological recovery may suffer long-term psychological sequelae, highlighting the need for psychological support in addition to neurological management (42).

The standardized assessment of post-traumatic neurological deficits is the cornerstone for effective communication, prognosis prediction, and treatment evaluation. Standardization in this field began in 1969 when Frankel et al. (47) pioneered the first internationally accepted five-grade (A–E) neurological function scale. This classification categorized injuries into complete, sensory only, motor non-useful, motor useful, and complete recovery, marking a transition from qualitative description to standardized evaluation in spinal cord injury assessment (Table 5).

**Table 5:** Comparison of Frankel Grade and ASIA Impairment Scale (AIS) for spinal cord injury (47, 48).

Grade/Scale	Frankel Grade (1969)	ASIA Impairment Scale (AIS) (1982 onwards)	Key distinction
A	Complete: No motor or sensory function preserved below the neurological level	Complete: No motor or sensory function is preserved in the sacral segments S4–S5	ASIA’s definition is stricter and anatomically precise, focusing on the presence or absence of sacral sparing as the absolute criterion
B	Sensory only: Sensory function preserved below the neurological level, but motor function is completely absent	Sensory incomplete: Sensory but not motor function is preserved below the neurological level and includes the sacral segments S4–S5	ASIA explicitly requires sacral sensation to be present to qualify as incomplete
C	Motor useless: Some motor function is preserved below the neurological level, but it is of no practical use to the patient	Motor incomplete: Motor function is preserved below the neurological level, and more than half of key muscle functions below the neurological level have a muscle grade less than 3	Frankel’s “useless” is a subjective, functional term. ASIA provides an objective, quantitative criterion based on the number of muscles with non-functional strength (<3/5)
D	Motor useful: Useful motor power is preserved below the neurological level; patients can move lower limbs and many can walk	Motor incomplete: Motor function is preserved below the neurological level, and at least half of key muscle functions below the neurological level have a muscle grade of 3 or greater	Frankel’s “useful” is also functional. ASIA again provides an objective, quantitative criterion based on the number of muscles with functional strength (≥3/5)
E	Recovery: Free of neurological symptoms (no weakness, no sensory loss). Abnormal reflexes may be present	Normal: Sensory and motor functions are graded as normal in all segments, and the patient had prior deficits	Definitions are largely similar, representing full neurological recovery

Although the Frankel scale was groundbreaking, its evaluation emphasized functional outcomes over precise anatomical localization, leading to a degree of subjectivity in grading (48). To improve assessment precision and prognostic reliability, the American Spinal Injury Association (ASIA) introduced the first edition of the International Standards for Neurological Classification of Spinal Cord Injury (ISNCSCI) in 1982, which has been continuously refined, with the latest major update published in 2019. A key refinement in ISNCSCI is its strict use of “sacral sparing”—defined as the presence of sensory or motor function in the S4–S5 dermatomes as the sole and absolute criterion for distinguishing between complete (AIS grade A) and incomplete (AIS grades B–E) injuries. This seemingly subtle definition, re-emphasized in the 2019 revision, carries major clinical implications: the initial determination of “incompleteness” (the transition from AIS A to B) hinges exclusively on sacral sensation or voluntary anal contraction, irrespective of motor function in the lower limbs (Table 5).

This seemingly subtle definition carries major clinical and scientific implications. A retrospective study of 804 patients with traumatic spinal cord injury directly compared the prognostic stability of the two grading systems. The study found that among patients initially classified as “incomplete” (grades B–D) using the Frankel system, 9.4% had neurological outcomes consistent with complete injury at one-year follow-up. In contrast, when using the ASIA classification (AIS B–E), which is based on sacral sparing, this proportion was only 2.0%. The study concluded that the ASIA classification provides a more stable and reliable prediction of final injury severity at initial evaluation, demonstrating stronger prognostic value in defining “incomplete” injury (49).

## 6 Artificial intelligence: the next step in classification

As this review has traced the evolution of classification systems toward improved reliability, the application of artificial intelligence (AI) represents the next logical paradigm shift in achieving these goals (50).

### 6.1 Automated detection and segmentation

In emergency settings, the rapid and accurate identification of injuries is critical (51). Deep learning models, such as convolutional neural networks (CNNs), excel at automating this process (52). These models can automatically segment vertebral structures and identify fracture lines with high sensitivity and specificity (53), achieving performance comparable or superior to human radiologists (54). This automation promises to optimize clinical workflows, reduce missed diagnoses, and ensure diagnostic consistency (55).

### 6.2 Automated classification and prognosis

AI’s greater value lies in its potential to resolve the core challenges of reliability and prognostication central to this review. Its primary application is automating complex systems like SLIC and AO spine. This directly confronts the inconsistent interobserver reliability of systems like SLIC, particularly in assessing discoligamentous complex (DLC) integrity, can be merely “moderate” or even “poor” (25). By training on large datasets, AI can apply classification rules with near-perfect consistency, eliminating the subjective discrepancies among clinicians.

Furthermore, machine learning algorithms are being used to build powerful prognostic tools (56). By integrating multi-dimensional data, these models combine morphological features from classifications with key clinical variables, such as the admission ASIA grade and MRI findings (57). This allows algorithms like support vector machines (SVM) or XG boost to predict long-term neurological recovery with high accuracy (58), enabling clinicians to provide data-driven prognoses and formulate more precise treatment strategies (59).

### 6.3 From algorithm to bedside

However, translating AI from algorithm to clinical practice faces key challenges. To overcome the “black box” problem and build clinical trust, algorithms must be interpretable. Explainable AI (XAI) techniques, such as saliency maps, can visualize the imaging evidence an AI used to make its classification (60). This transparency is fundamental for human-machine collaboration. Finally, rigorous, large-scale external validation is mandatory to ensure a model’s robustness across diverse hospitals, equipment, and populations, ensuring the leap from a technological revolution to clinical practice (61).

## 7 Summary and prospect

The evolution of cervical spine trauma classification systems marks a fundamental shift from descriptive morphology to functional assessment in service of clinical decision-making. Early systems were constrained by imaging and biomechanical knowledge, whereas modern classifications like the SLIC score and AO spine classification integrate three key elements: injury morphology, soft tissue integrity, and neurological function. This paradigm shift transforms classification from a descriptive science into an applied tool for guiding treatment. For the subaxial cervical spine, the SLIC system pioneered this integrated approach linked to treatment thresholds, while the AO spine classification refined it with more detailed morphological delineation to enhance reliability and accommodate complex injuries, providing a robust foundation for clinical practice.

Despite the success of current classifications, cervical spine trauma care is on the verge of a new technological revolution. Beyond artificial intelligence, advanced imaging is optimizing prognostication. Diffusion tensor imaging (DTI), by providing microstructural data, offers parameters like fractional anisotropy (FA) that correlate significantly with neurological injury severity (ASIA grade) and postoperative recovery potential (62). In the future, integrating this quantitative data with multi-parametric techniques assessing myelin and metabolism, such as quantitative magnetization transfer (qMT) and chemical exchange saturation transfer (CEST), promises a new paradigm for precision assessment that connects macro-imaging, micro-pathology, and clinical prognosis (63).

Finally, for patients with the poorest neurological prognosis (e.g., ASIA A), where acute-phase treatments offer limited functional gains, the treatment paradigm is shifting from “structural repair” to “functional reconstruction.” Brain-computer interface (BCI) technology offers new hope for these individuals (64). BCI is evolving from controlling external robotic arms to constructing “digital bridges” that directly modulate spinal cord neural circuits to restore a patient’s own motor functions. This technological trajectory not only provides functional compensation but also promotes long-term neuroplasticity, demonstrating immense potential for genuine neural repair (65).
